# Upregulation of Fatty Acid Synthase Increases Activity of β-Catenin and Expression of NOTUM to Enhance Stem-like Properties of Colorectal Cancer Cells

**DOI:** 10.3390/cells13191663

**Published:** 2024-10-08

**Authors:** Courtney O. Kelson, Josiane Weber Tessmann, Mariah E. Geisen, Daheng He, Chi Wang, Tianyan Gao, B. Mark Evers, Yekaterina Y. Zaytseva

**Affiliations:** 1 Department of Toxicology and Cancer Biology, University of Kentucky, Lexington, KY 40536, USA; courtney.kelson@uky.edu (C.O.K.); jo.tessman@uky.edu (J.W.T.); mge253@uky.edu (M.E.G.); 2 Biostatistics and Bioinformatics Shared Resource Facility, Markey Cancer Center, University of Kentucky, Lexington, KY 40536, USA; daheng.he@uky.edu (D.H.); chi.wang@uky.edu (C.W.); 3 Markey Cancer Center, University of Kentucky, Lexington, KY 40536, USA; tianyan.gao@uky.edu (T.G.); mark.evers@uky.edu (B.M.E.)

**Keywords:** colorectal cancer, fatty acid metabolism, FASN, Notum, stemness

## Abstract

Dysregulated fatty acid metabolism is an attractive therapeutic target for colorectal cancer (CRC). We previously reported that fatty acid synthase (FASN), a key enzyme of de novo synthesis, promotes the initiation and progression of CRC. However, the mechanisms of how upregulation of FASN promotes the initiation and progression of CRC are not completely understood. Here, using *Apc*/VillinCre and *Apc^Min^* mouse models, we show that upregulation of FASN is associated with an increase in activity of β-catenin and expression of multiple stem cell markers, including Notum. Genetic and pharmacological downregulation of FASN in mouse adenoma organoids decreases the activation of β-catenin and expression of Notum and significantly inhibits organoid formation and growth. Consistently, we demonstrate that NOTUM is highly expressed in human CRC and its expression positively correlates with the expression of FASN in tumor tissues. Utilizing overexpression and shRNA-mediated knockdown of FASN, we demonstrate that upregulation of FASN increases β-catenin transcriptional activity, NOTUM expression and secretion, and enhances stem-like properties of human CRC cells. Pharmacological inhibition of NOTUM decreases adenoma organoids growth and proliferation of cancer cells. In summary, upregulation of FASN enhances β-catenin signaling, increases NOTUM expression and stem-like properties of CRC cells, thus suggesting that targeting FASN upstream of the β-catenin/NOTUM axis may be an effective preventative therapeutic strategy for CRC.

## 1. Introduction

Colorectal cancer (CRC) is the second leading cause of cancer-related death for men and women combined in the United States [1]. In addition, the CRC rate is rising in young adults and is currently the leading cause of cancer-related death in men younger than 50 [2]. Thus, there is an urgent need to better understand the molecular mechanisms driving CRC so that effective new therapeutic strategies can be devised to prevent and combat this devastating disease.

Aberrant Wnt signaling is a common phenomenon observed in solid cancers, including CRC [3]. About 80% of the initiating events observed in the adenoma–carcinoma pathway of CRC development result from mutations inactivating APC, a vital tumor suppressor and major component of the destruction complex used for regulating the cytoplasmic pool of β-catenin [4]. The canonical Wnt/β-catenin pathway is well-known for regulating tissue homeostasis, proliferation, cell cycle, and stemness in the intestinal epithelium [5,6].

Altered fatty acid metabolism and overexpression of lipogenic enzymes are often observed in CRC [7,8]. Fatty acid synthase (FASN) is a vital enzyme of de novo lipid synthesis that utilizes acetyl-CoA and malonyl-CoA to produce palmitate [9,10]. Studies have shown that overexpression of FASN is associated with a poor clinical outcome, which provides a strong rationale to therapeutically target FASN in CRC [11,12]. Recently, our group demonstrated that FASN is highly upregulated during adenoma formation. Downregulation of this enzyme decreases the formation of adenomas and increases *Apc*/VillinCre mice survival [13]. Similar to canonical Wnt/β-catenin signaling, lipid metabolism contributes to the initiation and progression of cancer via tumor initiating cells, also known as cancer stem cells [14,15,16]. Furthermore, the enhancement of stem-like properties of these cancer stem cells to promote initiation and progression of CRC is attributed to the crosstalk between lipid metabolism and β-catenin signaling [16].

NOTUM is a carboxylesterase that removes palmitoleic acid from the Wnt ligands, decreasing their binding affinity for Wnt receptors, thus downregulating Wnt signaling [17,18]. Flanagan et al. demonstrated that intestinal *Apc*-mutant cells secrete Notum as a competitive strategy to outcompete wild-type cells and initiate CRC [19]. Single-cell analysis of transcriptomes from CRC patients identified NOTUM as one of the five-protein signatures of cancer stem cells that correlates with a poor prognosis in CRC [20]. Murine and human colorectal adenocarcinomas display NOTUM upregulation, suggesting its crucial involvement in CRC progression [21].

In this study, for the first time, we report that an increase in the expression of FASN upregulates the expression of NOTUM and other CRC stem cell markers in transgenic mouse models, mouse adenoma organoids, and human CRC cells. Consistently, we show a significant correlation between FASN and NOTUM expression in human CRC tissues. Genetic and pharmacological inhibition of FASN decreases β-catenin activity, NOTUM expression/secretion, and stem-like properties in CRC cells. Pharmacological inhibition of β-catenin decreases the expression of NOTUM and colony formation in CRC cells, suggesting that FASN regulates NOTUM via the β-catenin pathway and targeting FASN can be a preventative therapeutic strategy or early-stage treatment option for CRC.

## 2. Materials and Methods

### 2.1. Mouse Studies

Mice were housed at the facility supervised by the Division of Laboratory Animal Resources, University of Kentucky, in accordance with the NIH Guide for the Care and Use of Lab Animals (https://www.ncbi.nlm.nih.gov/books/NBK54050/) (accessed on 1 April 2024). All animal experimental procedures were carried out under approval from the University Committee on Use and Care of Animals, University of Kentucky, protocol #2016-2521 (PI: Zaytseva). *Apc*/VillinCre and *Apc*/Villin-CreERT2 mouse models with hetero- and homozygous deletion of *Fasn* were bred and established as previously described [13]. *Apc^Min^*/ERT2 and *Apc^Min^* *Fasn*^+/Δ^/ERT2 mouse colonies were established by mating *Apc^Min^* with ERT2 and *Fasn*^+/Δ^/ERT2 mice, respectively. Mouse models used in this study are shown in Supplementary Table S1. To induce deletion of *Apc* and/or *Fasn,* mice were injected with tamoxifen for 5 consecutive days (75 mg/kg body weight), and intestinal tissues were collected 10 days later. Intestinal tissues from mice without VillinCreERT2 or without the target gene injected with tamoxifen were used as a control. Intestinal adenoma tissues were collected and analyzed as described [13].

### 2.2. Organoid Culture

Mouse intestinal organoids were extracted and isolated as previously described [22] and cultivated in IntestiCult Organoid Growth Mouse Medium (#06005, StemCell Technologies, Vancouver, BC, Canada). Organoids were dissociated into single cells by TrypLE Express Enzyme (#12605010, ThermoFisher, Waltham, MA, USA) and re-plated on 24-well plates in 60 µL of Matrigel. Human organoids were established from freshly collected CRC tissues (IRB#52094, PI Zaytseva). Tumor tissues were washed in PBS and dissociated with Liberase (50 µg/mL). Dissociated tissues were put through different-size strainers to collect 40–100 µm organoids. The collected organoids were washed, resuspended in Matrigel, and plated. Organoids were cultured on IntestiCult™ Organoid Growth Medium (#06010, StemCell Technologies, Vancouver, BC, Canada).

### 2.3. Cell Culture and Transfection

Established cell lines, HT29 and HCT116, were maintained in McCoy’s 5A medium, and SW480 was maintained in Dulbecco’s Modified Eagle’s Medium (DMEM) supplemented with 10% FBS (Sigma-Aldrich, St. Louis, MO, USA) and 1% penicillin–streptomycin. The cells were cultivated in a humidified atmosphere containing 5% CO_2_ at 37 °C. Stable FASN knockdown HT29 and HCT116 cells were established from FASN shRNAs from Sigma-Aldrich and pLKO.1-puro non-mammalian shRNA was used as a non-target control (NTC). The following shRNA was used to knockdown FASN: TRCN0000003125 (FASNsh#1), TRCN0000003127 (FASNsh#2), and TRCN0000003129 (FASNsh#3). Cells were transfected with 8 μg/mL of polybrene and selected with 10 μg/mL of puromycin. Stable FASN overexpression was established by transfecting SW480 cells with an enhanced green fluorescent protein (PEGFP)-FASN vector with gentamicin (Invitrogen, Austin, TX, USA) as previously described [23]. FASN knockdown and overexpression were confirmed by real-time qPCR and western blot analyses.

### 2.4. Real-Time Reverse Transcription-Polymerase Chain Reaction (RT-PCR) Analysis

Total RNA was isolated using an RNeasy mini kit (Qiagen, Germantown, MD, USA). cDNA was synthesized using a high-capacity cDNA reverse transcription kit (Applied Biosystems, Bedford, MA, USA; #4368814). qRT-PCR was carried out using a TaqMan Gene Expression Master Mix (Applied Biosystems; #4369016) according to the manufacturer’s protocol and TaqMan probes for human FASN (#4331182-Hs01005622_m1), NOTUM (#4331182-Hs00394510_m1), ALCAM (#4331182-Hs00977641_m1), PROM1 (#4331182-Hs01009259_m1), CD44 (#4331182-Hs01075864_m1), and GAPDH (#4331182-Hs02786624_g1) and mouse *Fasn* (#4331182-Mm00662319_m1), *Notum* (#4331182-Mm01253273_m1), *Lgr5* (#4331182-Mm00438890_m1), *Alcam* (#4331182-Mm00711623_m1), *Prom1* (#4331182-Mm01211402_m1), *CD44* (#4331182-Mm01277161_m1), and *Gapdh* (#4331182-Mm99999915_g1) from ThermoFisher.

### 2.5. Protein Extraction and Western Blot Analysis

Mouse tissues, organoids, and CRC cells were lysed using Cell Lysis Buffer (#9803, Cell Signaling, Danvers, MA, USA) supplemented with protease inhibitor cocktail (#11836170001, Roche, Indianapolis, IN, USA) and 1 mM PMSF (#93482, Sigma). The membranes were probed with the following primary antibodies: fatty acid synthase (Cell Signaling #3180), Notum (#106448, Abcam, Waltham, MA, USA), active β-catenin (Cell Signaling #7270), ALCAM/CD166 (Abcam #109215), CD133 (Biorbyt #10288, Durham, NC, USA), CD44 (#156-3C11, Cell Signaling), cyclin D1 (#ab134175, Abcam), MUC2 (#CCP58, Cell Signaling), and β-actin (#A5441, Abcam). Next, membranes were incubated with the appropriate secondary antibodies (rabbit #7074 or mouse #7076), and protein bands were detected with Immobilon Western Chemiluminescent HRP substrate (#WBKLS0500, Sigma). The visualization of band intensities was performed using Adobe Photoshop software version # 25.3.1. The quantification of band intensities was performed via ImageJ software version 1.53.

### 2.6. T-Cell Factor/Lymphoid Enhancer Factor (TCF/LEF) Reporter Luciferase Assay

The canonical Wnt/β-catenin signaling activity was measured by using a TCF/LEF reporter kit (BPS Biosciences, San Diego, CA, USA). Both SW480 (control and FASN overexpressed) and HT29 cells were seeded at 20,000 cells per well in a 96-well clear bottom plate. The next day, the cells were transfected with TCF/LEF luciferase reporter vector. After 24 h, the media was replaced and followed by 0.2 µM TVB-3664 for HT29 cells. After 48 h, SW480 and HT29 cells were lysed and analyzed for luciferase activity using a Dual-luciferase reporter assay kit (Promega).

### 2.7. Inhibitors

The following inhibitors were utilized for FASN, active β-catenin, and Notum, respectively: TVB-3664 (C_25_H_23_F_3_N_4_O_2_), 3V-Biosciences (Menlo Park, CA, USA); MSAB (C_15_H_15_NO_4_S), Selleck Chemicals (Houston, TX, USA); LP-922056 (C_11_H_9_CIN_2_O_2_S_2_), Tocris Bioscience (Minneapolis, MN, USA); Caffeine, APExBIO (Houston, TX, USA).

### 2.8. Spheroid Formation Assay

HT29 and HCT116 cells were grown in suspension in low serum media with additive growth factors and appropriate inhibitors as previously described [24]. After 7–10 days, wells were imaged and spheroids larger than 50 µm were counted/scored. The following formula was used to calculate the spheroid formation efficiency: number of scored spheroids/seeding density × 100 [25].

### 2.9. Tumor Organoid Colony Formation Assay

Tumor organoids generated from *Apc^Min^*/*Fasn*^+/Δ^/ERT2 and *Apc^Min^* mice were cultured as previously described [26]. To determine the effect of genetic or pharmacological inhibition of FASN on colony formation capability, single cell suspensions of tumor organoids were created using TrypLE Express Enzyme (ThermoFisher #12605010) for 10 min. A total of 2000 cells were embedded in 60 µL Matrigel and treated with 0.1 µM 4-OHT (genetic inhibition) or 0.2 µM TVB-3664 (pharmacological inhibition) in organoid media for 24 h. The media was subsequently changed. The number of tumor organoids formed after 5 days was counted under an inverted microscope.

### 2.10. Click-iT Plus EdU Imaging Analysis

*Apc^Min^* organoids were digested and plated as single cells in 20 µL Matrigel on an 8-well coverslip μ-slide (#80807, Ibidi, Fitchburg, WI, USA). Organoids were treated with 45 µM LP-922056 (Lexicon Pharmaceuticals, The Woodlands, TX, USA), 0.2 µM TVB-3664 (3V-Biosciences), or 200 µM caffeine (Apexbio Technology, Houston, TX, USA) for 72 h, and the media was changed every day. After the third day, the organoids were incubated with 10 µM EdU solution for 2 h. Next, the organoids were fixed, permeabilized, and stained with Alexa Fluor and Hoechst according to the manufacturer’s protocol (ThermoFisher #C10638). Images were captured using a Nikon A1 Confocal Microscope (Nikon, Shinagawa District, Tokyo, Japan).

### 2.11. Human NOTUM ELISA

HCT116 control and FASNsh-RNA-mediated knockdown cells were plated on 24-well culture plates for 48 h. Subsequently, media was collected, centrifuged, and utilized to perform NOTUM ELISA according to the manufacturer’s protocol (#HUFI01515 AssayGenie, Dublin, Ireland). Then, 450 nm absorbance was measured using a microplate reader (Varioskan LUX, ThermoFisher). Notum concentrations were calculated and quantified from the standard curve.

### 2.12. Soft Agar Colony Formation Assay

The 6-well cell culture plates were first layered with 1% soft agar using SeaPlaque GTG Agarose (#50115, Lonza, Allendale, NJ, USA) melted in Milli-Q water. HT29 and SW480 cells were trypsinized, counted, and treated with 5 µM MSAB or 100–200 µM caffeine. Cells were mixed with a 0.5% soft agar and 2X McCoy’s 5A medium (HT29) or 2X DMEM medium (SW480) solution and plated at a concentration of 5000–10,000 cells/well. McCoy’s 5A or DMEM medium supplemented with 10% FBS was placed on top of both agarose layers, and the media levels were monitored. Colonies were allowed to grow for 14 days and then were stained with 0.01% crystal violet (#C6158-50G, MilliporeSigma, St. Louis, MO, USA) for 30 min at room temperature. Wells were then washed with Milli-Q water to remove the excess crystal violet stain, imaged, and the colony diameter measured.

### 2.13. Cell Viability Assay

HT29 and HCT116 cells were seeded in 2D or 3D cultures in 96-well plates at a concentration of 3 × 10^3^ cells per well. Cells were treated with 0.2 µM TVB-3664, followed by incubation at 37 °C for 7 days. Then, 100 µL of medium containing 100 µL Cell-Titer Glo 2.0 (#G9241, Promega, Madison, WI, USA) or 3D cell viability reagent (#G9681, Promega) was added to the wells, followed by 2 min shaking, and incubated for 10 min at room temperature, protected from light. The luminescence was measured using a microplate reader (Varioskan LUX, ThermoFisher). SW480, HT29, and HCT116 cells were seeded in 96-well plates at a concentration of 3 × 10^3^ cells per well and treated with various concentrations of MSAB or caffeine, followed by incubation at 37 °C for 3 days.

### 2.14. Statistical Analysis

The Biostatistics and Bioinformatics Shared Resource Facility assisted with RNAseq and The Cancer Genome Atlas Colon Adenocarcinoma (TCGA-COAD) data set analysis. Data from other experiments was analyzed using GraphPad Prism version 9.1.1. Comparisons between groups were performed using one-way ANOVA and Dunnett’s post-test. Statistical significance between NTC and FASNsh groups; control and TVB-treated groups were analyzed using a paired *t*-test.

## 3. Results

### 3.1. Hetero- and Homozygous Deletion of Fasn in Apc/VillinCre and Apc/VillinCre-ERT2 Mouse Models Decreases the Expression of Active β-Catenin and Stem Cell Markers

**Figure 1:**
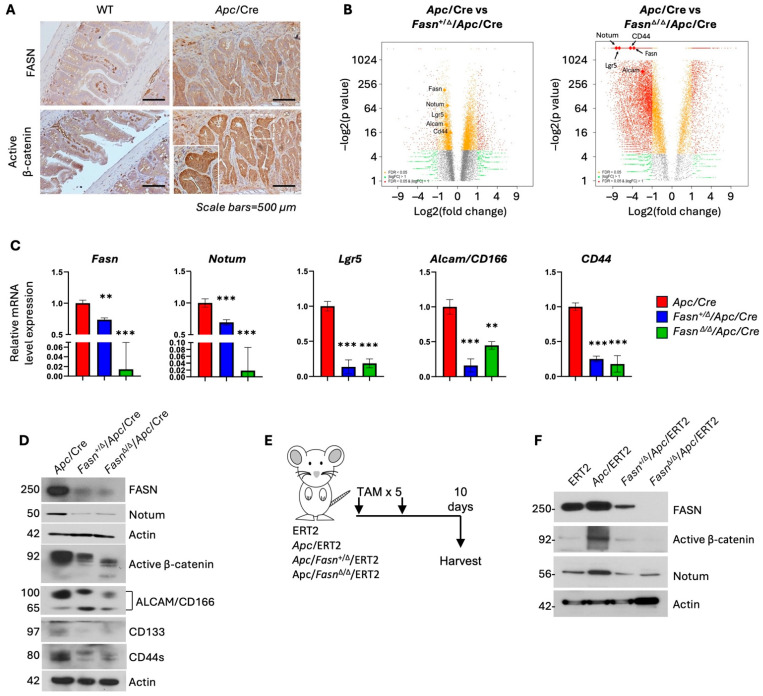
Hetero- and homozygous deletion of *Fasn* in intestinal tissues of *Apc*/Cre and *Apc*/ERT2 mice decreases the level of active β-catenin and expression of Notum. (**A**) FASN and active β-catenin expression in *Apc*/Cre mouse intestinal adenomas. (**B**) Volcano plots demonstrating the expression of stem cell markers, including Notum, in adenomas from *Apc*/Cre, *Apc*/*Fasn*^+/Δ^/Cre, and *Apc*/*Fasn*^Δ/Δ^/Cre mice. Yellow is FDR < 0.05; green is |logFC| > 1; red is FDR < 0.05 and |logFC| > 1. (**C**) mRNA levels of Fasn, Notum, and other stem cell markers in mouse intestinal adenomas using qRT-PCR analysis (*n* = 3; ** *p* < 0.01,*** *p* < 0.001, SEM). (**D**) Western blot for FASN, active β-catenin, and stem cell markers in *Apc*/Cre mouse intestinal adenomas with hetero- and homozygous deletion of *Fasn*. (**E**) Schematic timeline of tamoxifen (TAM) injections to induce deletion of *Apc* and *Fasn* in ERT2 models. Mice were injected with 75 mg of tamoxifen per 1 kg for 5 consecutive days. Mice were sacrificed, and tissue was collected and analyzed 10 days after the last injection. (**F**) Western blot for FASN, active β-catenin, and Notum expression in intestinal tissues after TAM injections.

We have previously reported that FASN promotes CRC initiation via upregulation of pro-carcinogenic signaling, including Wnt signaling pathways, in an *Apc*/VillinCre (*Apc*/Cre) mouse model [13]. Immunohistochemistry analysis shows that FASN and active β-catenin are significantly upregulated in adenomas from *Apc*/Cre mice compared to intestinal tissues from wild-type mice (Figure 1A). Volcano plots generated based on RNAseq analysis of intestinal adenomas from *Apc*/Cre, *Fasn*^+/Δ^/*Apc*/Cre, and *Fasn*^Δ/Δ^/*Apc*/Cre mice demonstrate that hetero- and homozygous deletion of *Fasn* is associated with decreased expression of CRC stem cell markers *Lgr5*, *Notum*, *CD44*, and *ALCAM/CD166* (Figure 1B, Supplementary Table S2). These findings were confirmed by qRT-PCR (Figure 1C) and western blot analyses (Figure 1D) on adenomas from *Apc*/Cre, *Fasn*^+/Δ^/*Apc*/Cre, and *Fasn*^Δ/Δ^/*Apc*/Cre mice. To further confirm these findings and ensure that FASN-mediated changes in active β-catenin and Notum are not restricted to the *Apc*/Cre mouse model, we utilized the inducible *Apc*/VillinCre-ERT2 (*Apc*/ERT2) model in which *Apc* and *Fasn* deletions are induced in adult mice via tamoxifen injections (Figure 1E). As shown in Figure 1F, *Apc* deletion in the intestinal tissues of adult mice increased the expression of FASN along with active β-catenin and Notum. Similar to the *Apc*/Cre model, *Fasn* deletion led to a decrease in active β-catenin and Notum expression, suggesting that active β-catenin and Notum are regulated by FASN in this model as well (see Figure 1F). Together, data from mouse models of *Apc*-driven carcinogenesis suggest that FASN expression is upregulated due to *Apc* loss and increases expression of active β-catenin and Notum in intestinal tissue and adenomas.

### 3.2. Downregulation of Fasn Decreases Expression of Active β-Catenin and Notum and Inhibits Organoid Growth and Stemness in ERT2-Inducible Apc^Min^ Organoid Models

**Figure 2:**
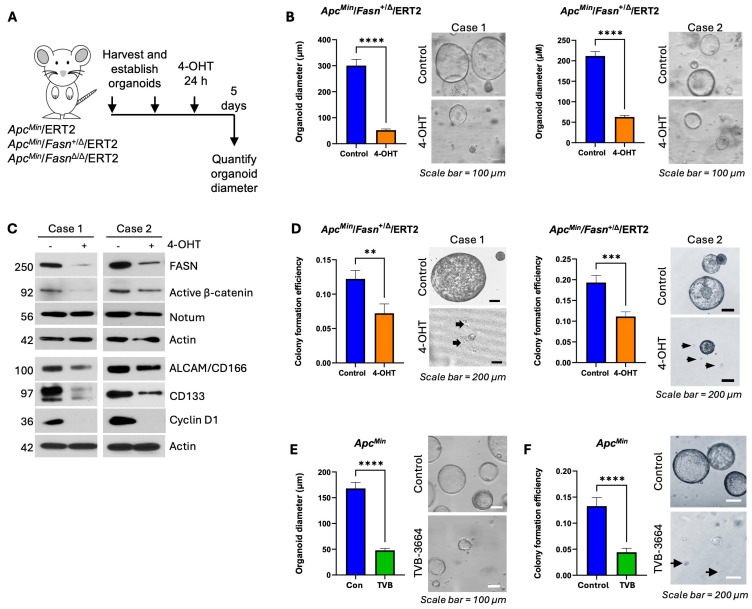
FASN promotes organoid formation and growth via upregulation of active β-catenin, Notum, other stem cell markers, and Cyclin D1 in ERT2-inducible *Apc^Min^* organoid models. (**A**) Schematic timeline of 4-hydroxytamoxifen (4-OHT) treatments to induce deletion of *Apc* and *Fasn* in *Apc^Min^*/*Fasn*^+/Δ^/ERT2 organoids. Images of organoids treated with 1 μM 4-OHT for 24 h and imaged and quantified on day 5. (**B**) Quantification and representative images of organoid size from control and 4-OHT *Apc^Min^*/*Fasn*^+/Δ^/ERT2 organoids. Number of organoids measured: Case 1 (*n* = 18 control organoids, *n* = 18 4-OHT-treated organoids), Case 2 (*n* = 30 control organoids, *n* = 31 4-OHT-treated organoids). (**C**) Western blot for FASN, active β-catenin, Notum, and stem cell markers in cases 1 and 2 of control and 4-OHT *Apc^Min^/Fasn*^+/Δ^/ERT2 organoids. (**D**) Quantification and representative images of tumor organoid colony formation assay in cases 1 and 2 of control and 4-OHT *Apc^Min^/Fasn*^+/Δ^/ERT2 organoids. Black arrows indicate single cells prevalent in treated organoids. (**E**) Quantification and representative images of organoid size from control and TVB-treated *Apc^Min^* organoids. *n* = 87 control organoids, *n* = 117 TVB-treated organoids. (**F**) Quantification and representative images of colony formation from control and TVB-treated *Apc^Min^* organoids. (*n* = 26 control organoids, *n* = 35 TVB-treated organoids). All functional assays were performed at least two or three times with multiple replicates. Representative data are shown. ** *p* < 0.01; *** *p* < 0.001; **** *p* < 0.0001, SEM.

To evaluate the impact of FASN on stemness, we utilized the inducible *Apc^Min^* organoid model. We have established organoids from the adenomas of *Apc^Min^*/ERT2 and *Apc^Min^*/*Fasn*^+/Δ^/ERT2 mice as previously described [27]. To induce *Fasn* deletion, we treated organoids with 1 µM of 4-hydroxytamoxifen (4-OHT) for 24 h. As shown in Supplementary Figure S1A,B, 1 µM of 4-OHT treatment does not affect organoid growth. In 5 days, we quantified and analyzed the size of the organoids (Figure 2A). Consistent with the published study on the role of FASN in anchorage-independent growth [28], we found that ERT2-induced deletion of *Fasn* leads to a significant decrease in organoid size in *Apc^Min^*/*Fasn*^+/Δ^/ERT2 organoids (Figure 2B). Western blot analyses on organoids show that heterozygous deletion of *Fasn* leads to a decrease in the expression of active β-catenin, Notum, ALCAM, CD133, and Cyclin D1, a downstream target of Wnt/β-catenin. (Figure 2C). Supplementary Figure S2 demonstrates that significant downregulation of *Fasn* mRNA expression in *Apc^Min^*/*Fasn*^+/Δ^/ERT2 organoids corresponds to a significant decrease in *Notum* mRNA. Heterozygous deletion of *Fasn* also leads to inhibition of colony formation as assessed by the tumor organoid colony formation assay (Figure 2D). To test the effect of pharmacological inhibition of FASN, we utilized TVB-3664 (TVB), a potent inhibitor against FASN enzymatic activity [29]. Consistent with genetic downregulation of *Fasn,* TVB significantly decreased *Apc^Min^* organoid size (Figure 2E) and colony forming ability of *Apc^Min^* organoids (Figure 2F). Collectively, these data suggest that *Fasn* is required for mouse adenoma organoid formation and growth in the inducible *Apc^Min^* organoid model.

### 3.3. NOTUM Is Overexpressed and Positively Correlates with FASN Expression in Human CRC

**Figure 3:**
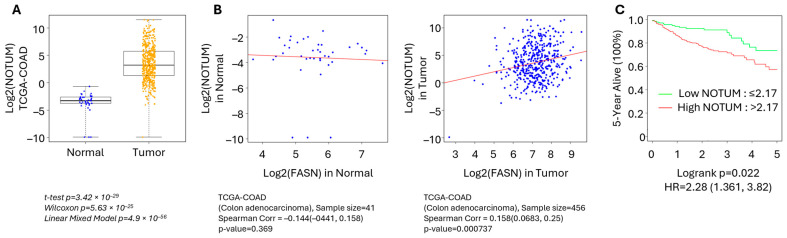
NOTUM is overexpressed and positively correlates with FASN expression in human CRC. (**A**) NOTUM mRNA expression in colorectal cancer patient samples from the TCGA-COAD mRNA dataset (*n* = 41 normal and *n* = 480 tumor samples). (**B**) Correlations between NOTUM and FASN mRNA expression were determined in normal tissues and CRC adenocarcinomas based on the TCGA-COAD mRNA dataset. (**C**) The 5-year survival analysis of CRC patients based on NOTUM expression using the TCGA-COAD dataset.

To investigate whether our findings on the FASN/NOTUM axis are relevant to human CRC, we utilized The Cancer Genome Atlas Colon Adenocarcinoma (TCGA-COAD) dataset to assess NOTUM mRNA expression in normal and CRC tissues. As shown in Figure 3A, NOTUM expression is significantly higher in CRC tissues as compared to normal mucosa. Interestingly, the Spearman correlation analysis shows no correlation between FASN and NOTUM expression in normal tissues, whereas a significant positive correlation can be seen between FASN and NOTUM in tumor tissues (Figure 3B). Consistent with these data, CRC patients with high NOTUM expression have a lower 5-year survival compared to patients with higher NOTUM expression (Figure 3C). Together, these data demonstrate that NOTUM is overexpressed and positively correlates with FASN expression in CRC, leading to a significant decrease in survival for patients with high NOTUM expression.

### 3.4. FASN Upregulates β-Catenin Activity and Expression of NOTUM to Promote Stem-like Properties of CRC Cells

**Figure 4:**
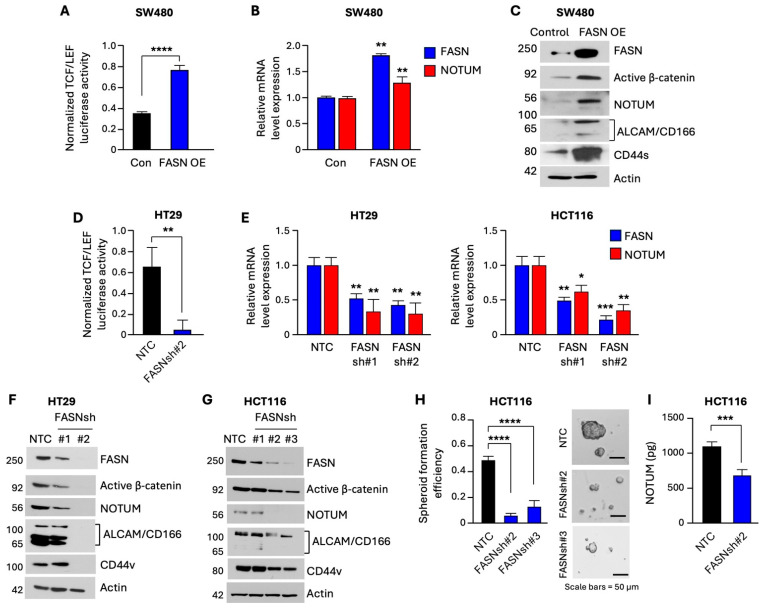
Overexpression of FASN increases β-catenin activity and expression of NOTUM to promote stemness in CRC. (**A**) The TCF/LEF reporter luciferase assay in SW480 control and FASN overexpression (OE) cells. (**B**) qRT-PCR analysis of FASN and NOTUM in SW480 control and FASN OE cells (*n* = 3). (**C**) Western blot analysis of FASN, active β-catenin, NOTUM, and other stem cell markers in SW480 control and FASN OE cells. (**D**) TCF/LEF luciferase assay in HT29 control and shRNA-mediated FASN knockdown cells. (**E**) qRT-PCR analysis of FASN and NOTUM in HT29 and HCT116 control and shRNA-mediated FASN knockdown, respectively (*n* = 3). (**F**,**G**) Western blot analysis of NOTUM and other stem cell markers in HT29 and HCT116 cells, respectively. (**H**) Quantification and representative images of spheroid formation in HCT116 control and FASNsh-RNA-mediated knockdown cells (*n* = 3). (**I**) Quantification of NOTUM secretion in HCT116 control and FASNsh-RNA-mediated knockdown cells (*n* = 3). * *p* < 0.05, ** *p* < 0.01, *** *p* < 0.001, **** *p* < 0.0001, SEM.

To investigate the role of FASN in human CRC via the potential crosstalk between β-catenin and NOTUM, we utilized HT29 (Apc mutant), SW480 (Apc mutant), and HCT116 (Apc wild-type, mutant for β-catenin) cell lines (Supplementary Table S3). HT29 and HCT116 contain high endogenous levels of FASN, whereas SW480 contains low levels, which is consistent with our previous findings [23]. To assess the functional consequence behind FASN overexpression (FASN OE) on β-catenin function, we utilized SW480 cells with overexpression of FASN. The TCF/LEF reporter luciferase assay demonstrates that FASN overexpression leads to an increase in β-catenin transcriptional activity (Figure 4A) and increases NOTUM mRNA expression (Figure 4B). Expression of active β-catenin and NOTUM was also increased at the protein level (Figure 4C). Consistent with our findings in mouse models, overexpression upregulated CD44 and CD166 in SW480 cells (see Figure 4C). The TCF/LEF luciferase assay for HT29 cells, with genetic inhibition of FASN via shRNA-mediated knockdown, showed that downregulation of FASN decreases β-catenin transcriptional activity (Figure 4D). Next, we sought to assess the effect of FASN inhibition on β-catenin signaling/activity and NOTUM expression. Knockdown of FASN led to a significant decrease in mRNA (Figure 4E) and protein levels (Figure 4F,G) of active β-catenin and NOTUM and other stem cell markers in HT29 and HCT116 cells. Additionally, knockdown of FASN decreased stem-like properties (Figure 4H) and NOTUM secretion in HCT116 cells (Figure 4I). These data suggest that FASN regulates stem-like properties and the expression of NOTUM and other stem cell markers via β-catenin signaling/activity.

### 3.5. Pharmacological Inhibition of FASN Decreases β-Catenin Activity, NOTUM Expression, and Stem-like Properties of CRC Cells

**Figure 5:**
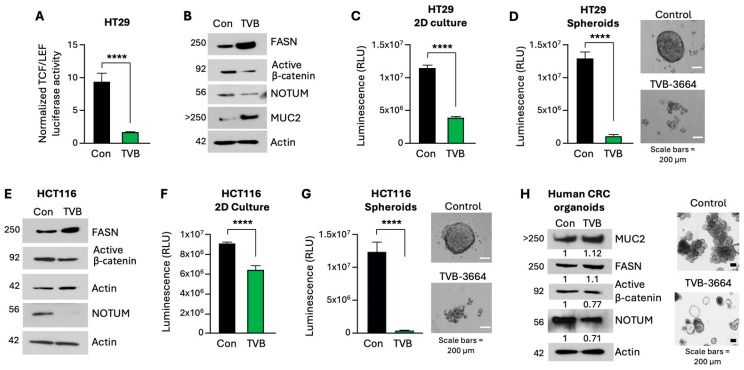
Pharmacological inhibition of FASN decreases β-catenin activity, NOTUM expression, and stemness of CRC cells. (**A**) TCF/LEF luciferase assay performed in HT29 control and TVB-treated cells. (**B**) Western blot analysis of FASN, NOTUM, active β-catenin, and MUC2 in HT29 control and TVB-treated cells. (**C**) Quantification of Cell-Titer Glo 2.0 cell viability of HT29 control and TVB-treated cells cultured in 2D. (**D**) Quantification and representative images of Cell-Titer Glo 3D cell viability of HT29 control and TVB-treated cells cultured in 3D. (**E**) Western blot analysis of FASN, NOTUM, and active β-catenin in HCT116 control and TVB-treated cells. (**F**) Quantification of Cell-Titer Glo 2.0 cell viability in 2D culture of control and TVB-treated HCT116 cells. (**G**) Quantification and representative images of Cell-Titer 3D cell viability in 3D culture of control and TVB-treated HCT116 spheroids. (**H**) Western blot on human organoids established from resected CRC and treated with TVB. Representative pictures of organoids at the time of collection (day 6) are shown. All functional assays were performed at least 3 times with multiple replicates. **** *p* < 0.0001, SEM.

Next, we determined the effect of pharmacological inhibition of FASN on β-catenin activity, expression of NOTUM and other stem cell markers, and stemness. Using the TCF/LEF luciferase assay, we show that FASN inhibition using TVB also leads to a significant decrease in β-catenin transcriptional activity in HT29 cells (Figure 5A). Consistent with our previous findings, western blot analysis shows an increase in the expression of FASN in response to TVB treatment due to inhibition of its enzymatic activity [30] (Figure 5B). TVB treatment decreases the levels of NOTUM and active β-catenin, whereas the expression of MUC2, an intestinal differentiation marker, is increased, suggesting that TVB decreases stemness and potentially promotes differentiation (see Figure 5B). Additionally, we found that TVB significantly decreases cell viability in 2D (Figure 5C) and 3D cultures (Figure 5D) of HT29 cells. Consistently, upon treatment with TVB, the expression of active β-catenin and NOTUM is decreased, whereas FASN expression is upregulated in HCT116 cells (Figure 5E). Similar to the results from HT29 cells, pharmacological inhibition of FASN significantly decreases viability of HCT116 cells in 2D (Figure 5F) and 3D cultures (Figure 5G). To further evaluate the effect of TVB in a clinically relevant model, we established human CRC organoids (see Methods). CRC organoids were treated with TVB for 6 days. Similar to the results from HT29 and HCT116, TVB decreased the expression of active β-catenin and NOTUM (Figure 5H; Supplementary Figure S3). Interestingly, TVB upregulates MUC2 expression, which corroborates the data from HT29 cells treated with TVB. Together, these data suggest that pharmacological inhibition of FASN decreases β-catenin activity, NOTUM expression, and stem-like properties of CRC human cells and organoids.

### 3.6. Inhibition of the β-Catenin/NOTUM Axis Leads to a Decrease in Proliferation of Adenoma Organoids and CRC Cells

**Figure 6:**
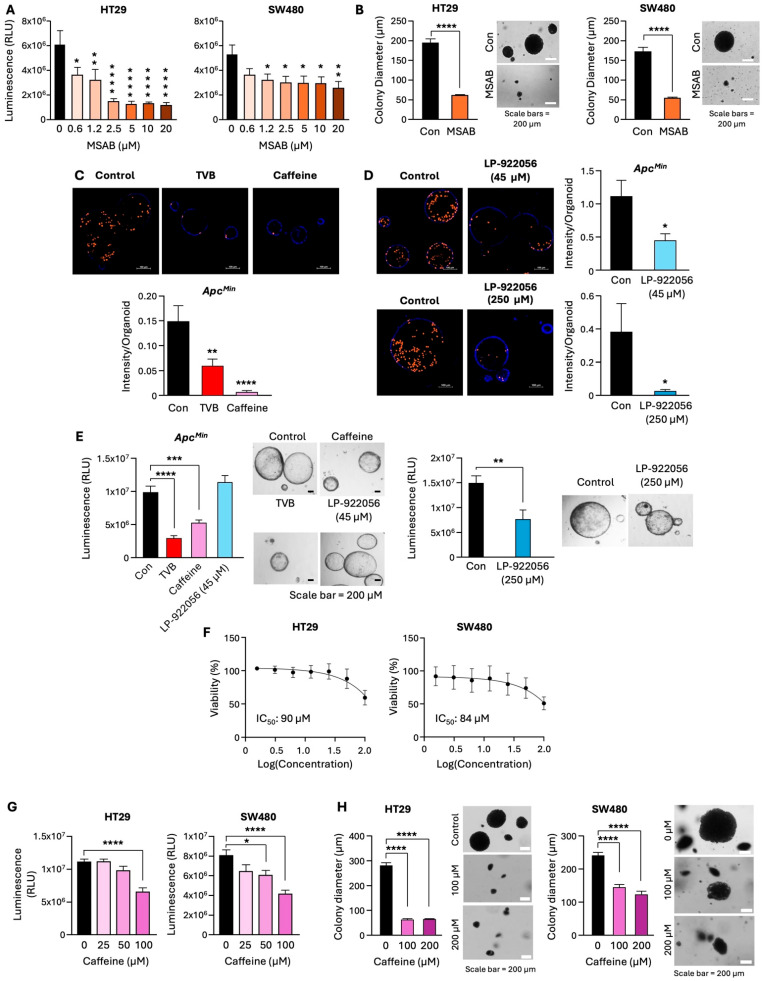
Inhibition of the β-catenin/NOTUM axis leads to a decrease in proliferation in CRC. (**A**) Quantification of viability in HT29 and SW480, control and MSAB-treated cells. (**B**) Quantification and representative images of colony formation in CRC cells treated with 5 µM MSAB. (**C**,**D**) Click-iT EdU cell proliferation assay. Representative images and quantification of DNA synthesis in *Apc^Min^* organoids treated with TVB (0.2 µM) and caffeine (200 µM) (**C**) and LP-922056 (45 µM and 250 µM) (**D**,**E**) Quantification and representative images of viability in *Apc^Min^* organoids treated with TVB (0.2 µM), caffeine (200 µM), and LP-922056 (45 µM and 250 µM). (**F**) CellTiter-Glo 3D cell viability assay. IC_50_ for caffeine in CRC cells. (**G**) Quantification of viability in HT29 and SW480 treated with various concentrations of caffeine. (**H**) Quantification and representative images of colony formation in HT29 and SW480 treated with caffeine. * *p* < 0.05; ** *p* < 0.01; *** *p* < 0.001; **** *p* < 0.0001, SEM.

To determine whether FASN regulates cellular proliferation via β-catenin/NOTUM, we utilized pharmacological approaches. MSAB, a β-catenin inhibitor, binds directly to β-catenin, promoting its proteasomal degradation [31]. MSAB treatments led to a significant decrease in cell viability (Figure 6A) and colony formation (Figure 6B) in HT29 and SW480 cells, confirming the pro-oncogenic role of β-catenin in CRC cells. Downregulation of active β-catenin by MSAB is confirmed by western blot (Supplementary Figure S4A). To assess the effect of pharmacological inhibition of NOTUM, we utilized LP-922056, a potent NOTUM inhibitor [19], and caffeine, which directly binds to the catalytic pocket of NOTUM, to inhibit its catalytic activity [32]. The previous work showed that treatment with 200 µM of caffeine leads to targeted inhibition of NOTUM activity in mouse organoids and cells [33]. We showed that TVB, caffeine, and LP-922056 (45 and 250 µM) treatment significantly inhibit the DNA synthesis and, thus, proliferation of *Apc^Min^* organoids (Figure 6C,D). Furthermore, we showed that TVB, caffeine, and LP-922056 (250 µM) significantly decrease the viability of *Apc^Min^* organoids (Figure 6E). Because of caffeine’s profound effect in *Apc^Min^* organoids, we sought to further investigate its effect on CRC cells. We identified the IC_50_ of caffeine in HT29 (90 µM) and SW480 (84 µM) and demonstrated that caffeine treatments led to a significant decrease in cell viability (Figure 6F,G). Moreover, caffeine decreased 3D growth of HT29 and SW480 cells (Figure 6H). Consistently, siRNA-mediated downregulation of NOTUM led to a decrease in expression of Cyclin D in SW480 cells (Supplementary Figure S4B,C). Altogether, these data suggest that β-catenin/NOTUM inhibition, particularly via caffeine, decreases proliferation and, subsequently, viability in adenoma organoids and CRC cells.

## 4. Discussion

Targeting FASN has been widely explored as a therapeutic opportunity for many cancers, including CRC [11,30]. Despite multiple studies showing its role in CRC progression and metastasis, utilization of FASN as a target for early stages of CRC or as a preventive strategy has not been extensively explored. The study by Bueno et al. demonstrated the essential role of FASN in acquiring 3D growth properties during malignant transformation, thus, for the first time, establishing FASN as a potential target for cancer prevention studies [28,34]. As a follow-up to this study, the investigation from our laboratory has demonstrated upregulation of *Fasn* expression during *Apc*-driven carcinogenesis and showed that *Fasn* promotes adenoma formation in the *Apc*/Cre mouse model, further suggesting its importance in CRC initiation [13]. However, the mechanisms of how *Fasn* promotes CRC initiation have not yet been investigated. In the current study, we show that upregulation of FASN in intestinal adenomas is associated with an increase in the levels of active β-catenin, Notum, and other stem markers such as Lgr5, Alcam, and CD44, which are associated with CRC. We show that downregulation of *Fasn* using genetic and pharmacological approaches in mouse *Apc*/Cre, *Apc*/ERT2, and *Apc^Min^* organoids leads to downregulation of these proteins and inhibits the formation and growth of adenoma organoids. These findings are consistent with studies on other cancers showing that pharmacological inhibition of FASN decreases stem-like properties in breast cancer cells [35] and downregulates the expression of stem cell markers in glioblastoma stem cells [36]. CRC stem cells uniquely reprogram lipid metabolism for their survival [37]. Some studies even suggest characterizing lipogenic enzymes such as FASN as cancer stem cell markers due to their strong affiliation with cancer stemness [38,39,40]. Our organoid models used in this study further support that *Fasn* is a vital contributor to stem-like properties such as organoid growth, formation, and expression of stem cell markers. Furthermore, pharmacological inhibition of FASN via TVB suggests that palmitate, a 16-carbon fatty acid and product of enzymatic activity of FASN, might be a potential mediator of FASN effects on stem-like properties of cells. Palmitate is an essential component in cancer cells, particularly for posttranslational modifications, signal transduction, energy production, and cell membrane maintenance [41]. FASN overexpression stabilizes cytoplasmic β-catenin via the palmitoylation of Wnt-1 in prostate cancer [42]. Consistently, our study shows that upregulation of FASN is associated with an increase in expression and activation of β-catenin in both mouse adenoma organoids and human CRC cells. In particular, we demonstrate that FASN inhibition downregulates the β-catenin/canonical Wnt signaling activity by decreasing its interaction with TCF/LEF, a specific transcription factor for β-catenin signaling [43]. This finding parallels a prior study demonstrating that the combination of FASN inhibition and taxane treatment decreases β-catenin expression in xenograft tumors [44]. Moreover, Wang et al. demonstrated that FASN regulates invasion and metastasis of CRC cells via upregulation of Wnt signaling [45].

NOTUM, a palmitoleoyl-protein carboxylesterase, is responsible for removing palmitoleic acid from Wnt proteins, decreasing their affinity to the Wnt receptors, and thus decreasing Wnt signaling in *Apc* wild-type crypt cells [19]. Flanagan et al. demonstrated that Notum secreted from mouse *Apc* mutant cells biases clonal competition to initiate cancer and that genetic and pharmacological inhibition of Notum prevents adenoma formation [19]. Consistently, a study by Yoon et al. showed that NOTUM is increased in plasma of patients with early-stage (rather than late-stage) CRC and upregulates proliferation and migration of CRC cells [46]. Additionally, NOTUM has been reported to inhibit stemness in gastric cancer [47]. Consistently, we show that downregulation of Notum, either via inhibition of FASN or directly, inhibits organoid and CRC cell proliferation and their stem-like properties, thus suggesting that targeting NOTUM can be a preventive strategy for CRC. Interestingly, pharmacological inhibition of FASN via TVB increases the expression of MUC2, a differentiation marker, in HT29 cells. The HT29 cell line is widely utilized in differentiation studies due to its high capacity to undergo differentiation [48,49,50,51]. In contrast, the HCT116 cell line has minimal capacity to undergo differentiation [48]; therefore, we did not see changes in expression of MUC2 in these cells. In both scenarios, we found that pharmacological inhibition of FASN decreases active β-catenin and NOTUM expression and stem-like properties of cancer cells. Importantly, we confirmed these findings in human CRC organoids, demonstrating a decrease in active β-catenin and NOTUM expression and an increase in MUC2 expression upon treatment with TVB, suggesting that inhibition of FASN with TVB decreases stemness and promotes differentiation in CRC.

Interestingly, recent studies suggest that NOTUM can be an effective target in advanced CRC [52]. Moreover, secreted NOTUM was found to increase the proliferation of SW480 and SW620 CRC cells [46]. In agreement, our study demonstrates high expression of NOTUM in human CRC and shows that pharmacological inhibition of NOTUM in CRC cells leads to a decrease in proliferation of cancer cells. Our study is the first to demonstrate that NOTUM inhibition via caffeine decreases the viability and stem-like properties of CRC cells. Additionally, our data suggest that caffeine is more efficacious in inhibiting proliferation and viability of *Apc^Min^* organoids than LP-922056, another commercially available inhibitor of NOTUM.

The recent study demonstrated that caffeine decreases the expansion of Apc mutant clones, but does not affect the growth, size, clonogenicity, or overall stemness of wild-type intestinal organoids [33]. Although our study highlights the autocrine effect of NOTUM inhibition on CRC cells and mouse tumor organoids, it does not investigate the NOTUM paracrine signaling in the initiation and progression of CRC, which is a weakness of this study and should be addressed in future investigations.

Recent studies have established that NOTUM inhibition can be a feasible approach to prevent and treat CRC, and multiple small molecular inhibitors of NOTUM are in development [17]. Our novel finding that inhibition of FASN leads to a significant downregulation of β-catenin activity as well as a decrease in secretion and expression of NOTUM provides another opportunity to target Wnt and NOTUM signaling. Our studies in mouse organoids and human cells are strongly supported by data in human specimens demonstrating a significant positive correlation between FASN and NOTUM expression in colorectal cancer patients. Furthermore, CRC patients with high NOTUM expression have a poor survival outcome compared to patients with low NOTUM expression, further corroborating a previous study [20].

## 5. Conclusions

Collectively, these data suggest that pharmacological inhibition of FASN decreases Wnt/β-catenin activity, NOTUM expression, and stem-like properties of adenoma organoids and CRC cells, suggesting that targeting FASN upstream of the β-catenin/NOTUM axis may be an effective therapeutic strategy for people at high risk of developing CRC as well as for CRC patients.

## Data Availability

All data included in the manuscript or in the Supplementary Materials are available upon request.

## Supplementary Materials

The following are available online at https://www.mdpi.com/article/10.3390/cells13191663/s1. Supplementary Table S1. Mouse models used in this study. Supplementary Table S2. Gene expression of stem cell markers in *Apc*/Cre and *Fasn*^Δ/Δ^ /*Apc*/Cre adenomas using RNAseq analysis. Supplementary Table S3. Mutational profile of CRC cell lines. Supplementary Figure S1. Treatment with 4-OHT does not significantly affect normal and tumor organoid growth. Supplementary Figure S2. *Fasn* and *Notum* mRNA expression in *Apc^Min^*/*Fasn*^+/Δ^/ERT2 organoids, control and treated with 4-OHT. Supplementary Figure S3. The effect of TVB-3664 treatment on growth of patient-derived CRC organoids. Representative images were taken daily. Supplementary Figure S4. The effects of beta-catenin and NOTUM inhibition.
