# Supplementary material for: Upregulation of Fatty Acid Synthase Increases Activity of β-Catenin and Expression of NOTUM to Enhance Stem-like Properties of Colorectal Cancer Cells
Source: Cells. 2024 Oct 8;13(19):1663. doi: 10.3390/cells13191663 (PMC11475157; doi:10.3390/cells13191663)
Supplement: Supplementary file 1 [file cells-13-01663-s001.zip › cells-3201475-supplementary.pptx]

## Slide 1
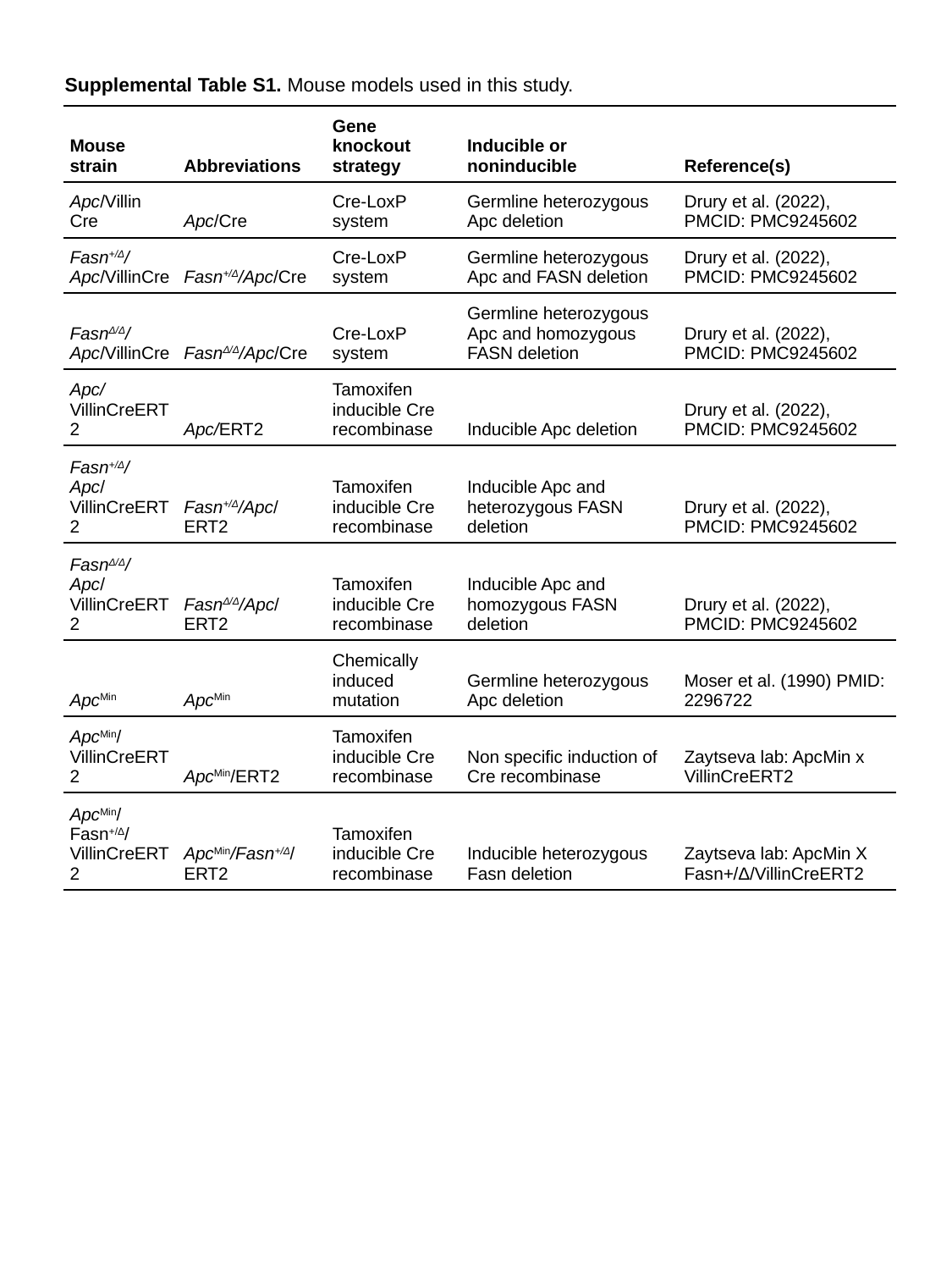

Supplemental Table S1. Mouse models used in this study.
| Mouse strain | Abbreviations | Gene knockout strategy | Inducible or noninducible | Reference(s) |
| --- | --- | --- | --- | --- |
| Apc/Villin Cre | Apc/Cre | Cre-LoxP system | Germline heterozygous Apc deletion | Drury et al. (2022), PMCID: PMC9245602 |
| Fasn+/Δ/Apc/VillinCre | Fasn+/Δ/Apc/Cre | Cre-LoxP system | Germline heterozygous Apc and FASN deletion | Drury et al. (2022), PMCID: PMC9245602 |
| FasnΔ/Δ/Apc/VillinCre | FasnΔ/Δ/Apc/Cre | Cre-LoxP system | Germline heterozygous Apc and homozygous FASN deletion | Drury et al. (2022), PMCID: PMC9245602 |
| Apc/VillinCreERT2 | Apc/ERT2 | Tamoxifen inducible Cre recombinase | Inducible Apc deletion | Drury et al. (2022), PMCID: PMC9245602 |
| Fasn+/Δ/Apc/VillinCreERT2 | Fasn+/Δ/Apc/ERT2 | Tamoxifen inducible Cre recombinase | Inducible Apc and heterozygous FASN deletion | Drury et al. (2022), PMCID: PMC9245602 |
| FasnΔ/Δ/Apc/VillinCreERT2 | FasnΔ/Δ/Apc/ERT2 | Tamoxifen inducible Cre recombinase | Inducible Apc and homozygous FASN deletion | Drury et al. (2022), PMCID: PMC9245602 |
| ApcMin | ApcMin | Chemically induced mutation | Germline heterozygous Apc deletion | Moser et al. (1990) PMID: 2296722 |
| ApcMin/VillinCreERT2 | ApcMin/ERT2 | Tamoxifen inducible Cre recombinase | Non specific induction of Cre recombinase | Zaytseva lab: ApcMin x VillinCreERT2 |
| ApcMin/Fasn+/Δ/VillinCreERT2 | ApcMin/Fasn+/Δ/ERT2 | Tamoxifen inducible Cre recombinase | Inducible heterozygous Fasn deletion | Zaytseva lab: ApcMin X Fasn+/Δ/VillinCreERT2 |

## Slide 2
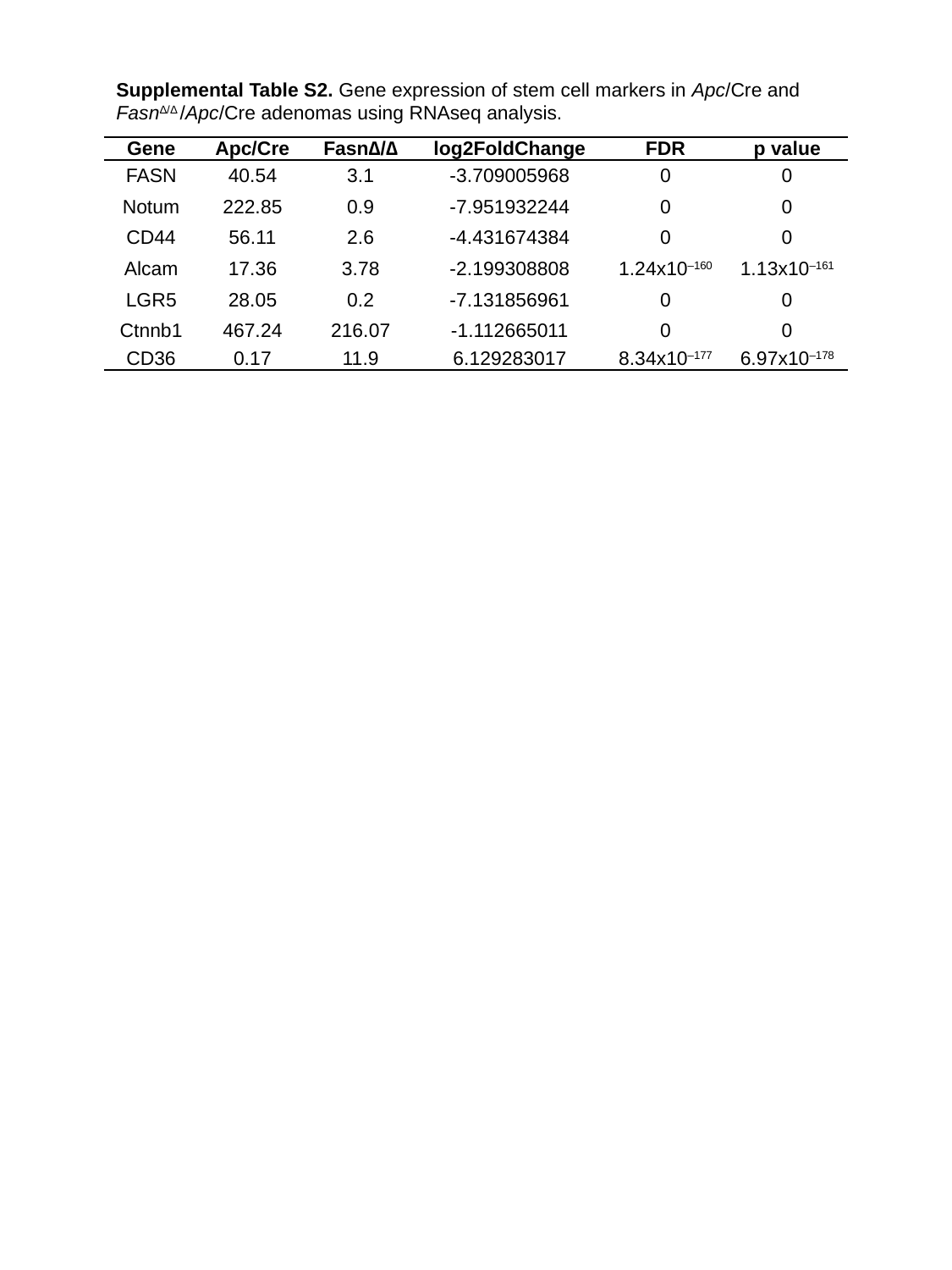

Supplemental Table S2. Gene expression of stem cell markers in Apc/Cre and FasnΔ/Δ /Apc/Cre adenomas using RNAseq analysis.
| Gene | Apc/Cre | Fasn∆/∆ | log2FoldChange | FDR | p value |
| --- | --- | --- | --- | --- | --- |
| FASN | 40.54 | 3.1 | -3.709005968 | 0 | 0 |
| Notum | 222.85 | 0.9 | -7.951932244 | 0 | 0 |
| CD44 | 56.11 | 2.6 | -4.431674384 | 0 | 0 |
| Alcam | 17.36 | 3.78 | -2.199308808 | 1.24x10–160 | 1.13x10–161 |
| LGR5 | 28.05 | 0.2 | -7.131856961 | 0 | 0 |
| Ctnnb1 | 467.24 | 216.07 | -1.112665011 | 0 | 0 |
| CD36 | 0.17 | 11.9 | 6.129283017 | 8.34x10–177 | 6.97x10–178 |

## Slide 3
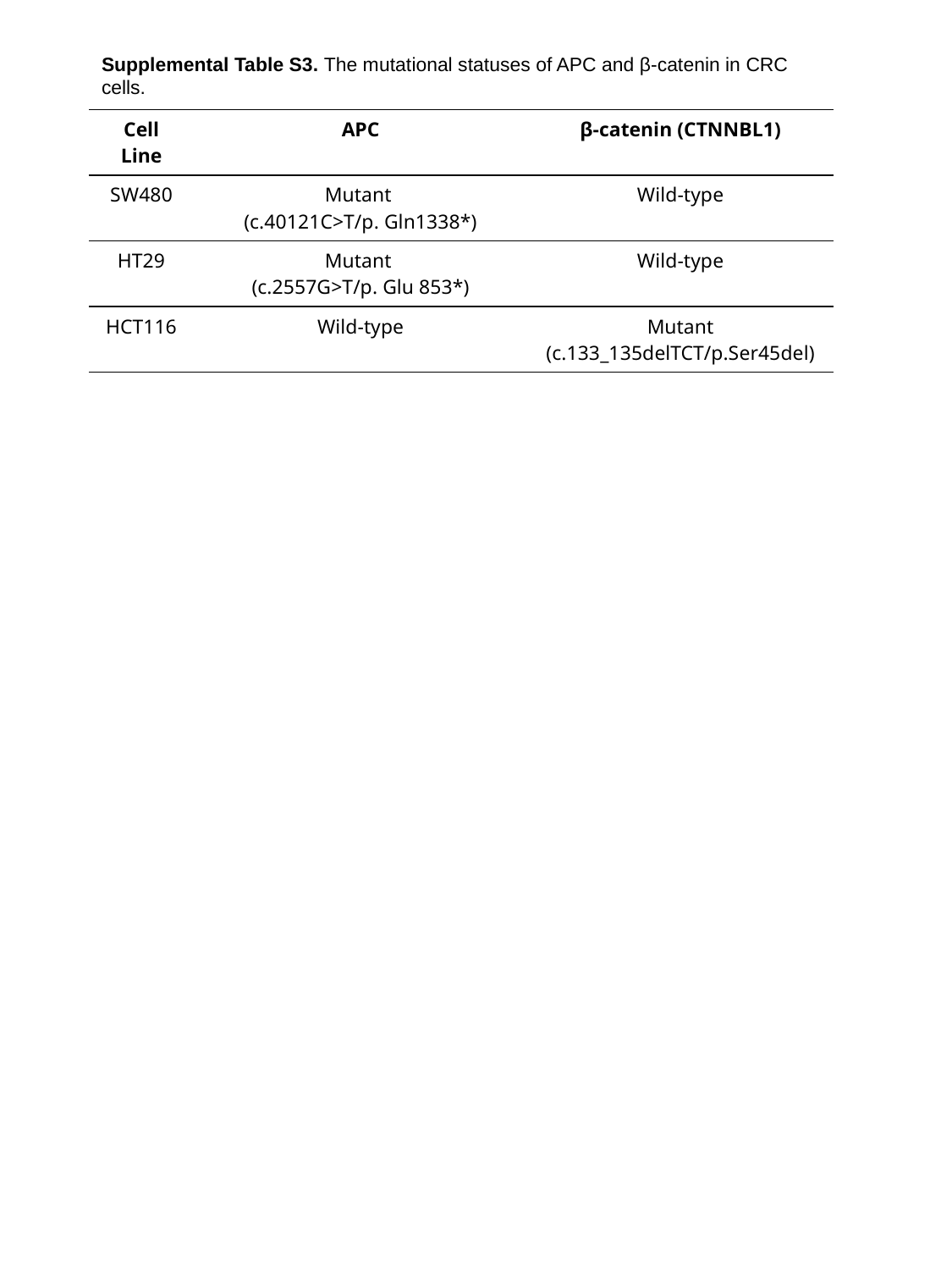

Supplemental Table S3. The mutational statuses of APC and β-catenin in CRC cells.
| Cell Line | APC | β-catenin (CTNNBL1) |
| --- | --- | --- |
| SW480 | Mutant (c.40121C>T/p. Gln1338\*) | Wild-type |
| HT29 | Mutant (c.2557G>T/p. Glu 853\*) | Wild-type |
| HCT116 | Wild-type | Mutant (c.133\_135delTCT/p.Ser45del) |

## Slide 4
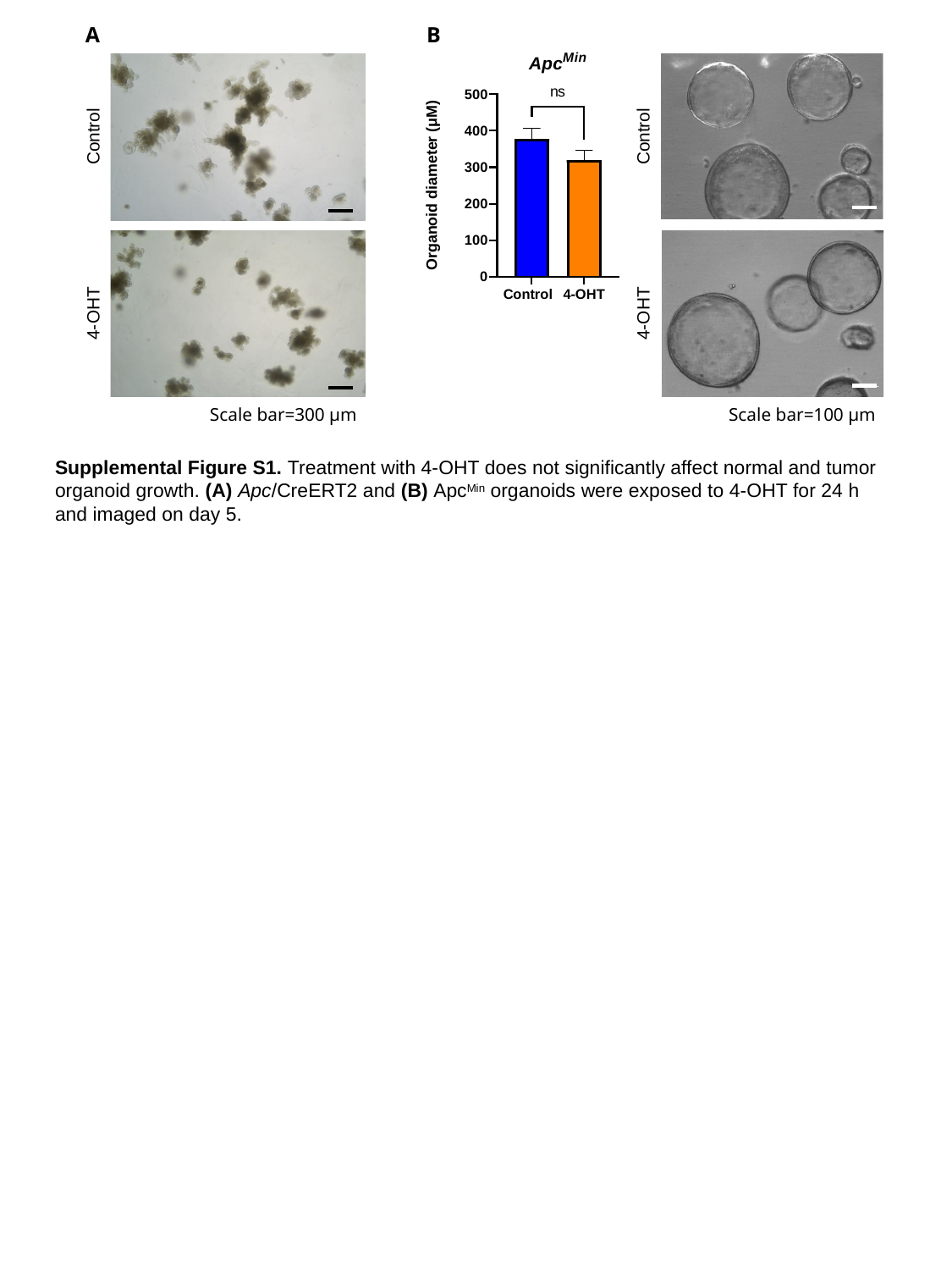

A
B
Control
Control
4-OHT
4-OHT
Scale bar=300 μm
Scale bar=100 μm
Supplemental Figure S1. Treatment with 4-OHT does not significantly affect normal and tumor organoid growth. (A) Apc/CreERT2 and (B) ApcMin organoids were exposed to 4-OHT for 24 h and imaged on day 5.

## Slide 5
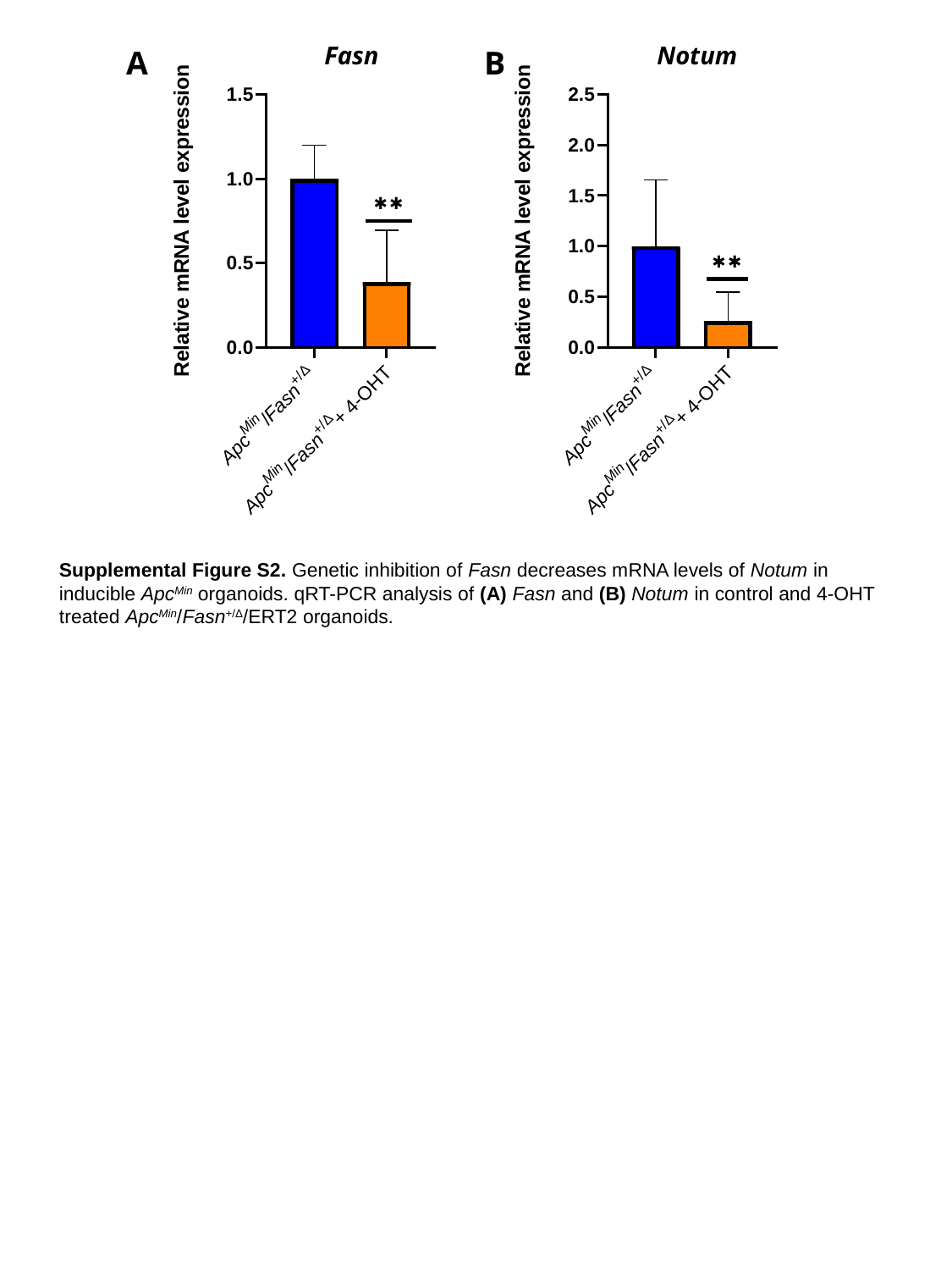

Fasn
Notum
A
B
Supplemental Figure S2. Genetic inhibition of Fasn decreases mRNA levels of Notum in inducible ApcMin organoids. qRT-PCR analysis of (A) Fasn and (B) Notum in control and 4-OHT treated ApcMin/Fasn+/Δ/ERT2 organoids.

## Slide 6
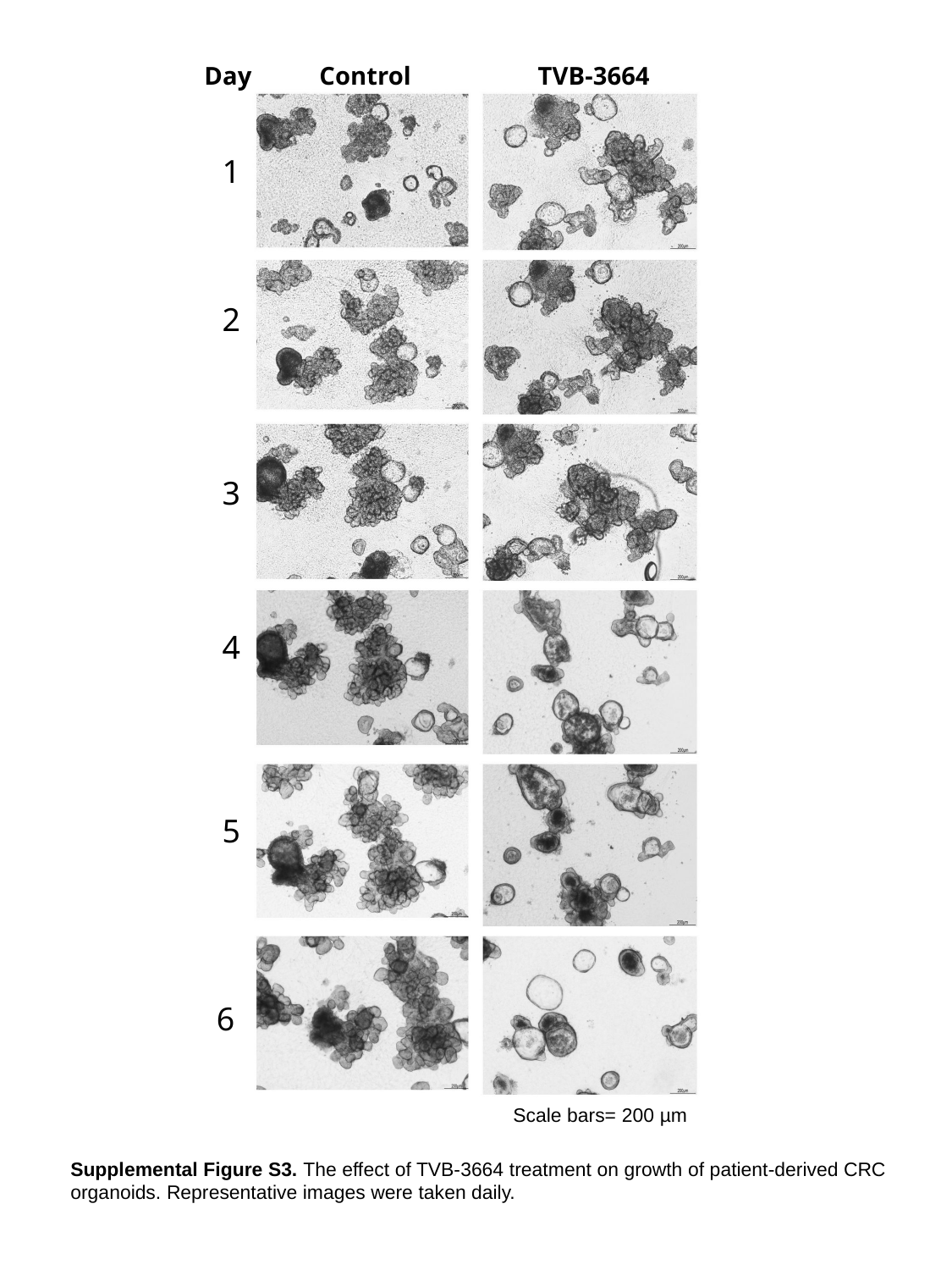

Day
Control
TVB-3664
1
2
3
4
5
6
Scale bars= 200 µm
Supplemental Figure S3. The effect of TVB-3664 treatment on growth of patient-derived CRC organoids. Representative images were taken daily.

## Slide 7
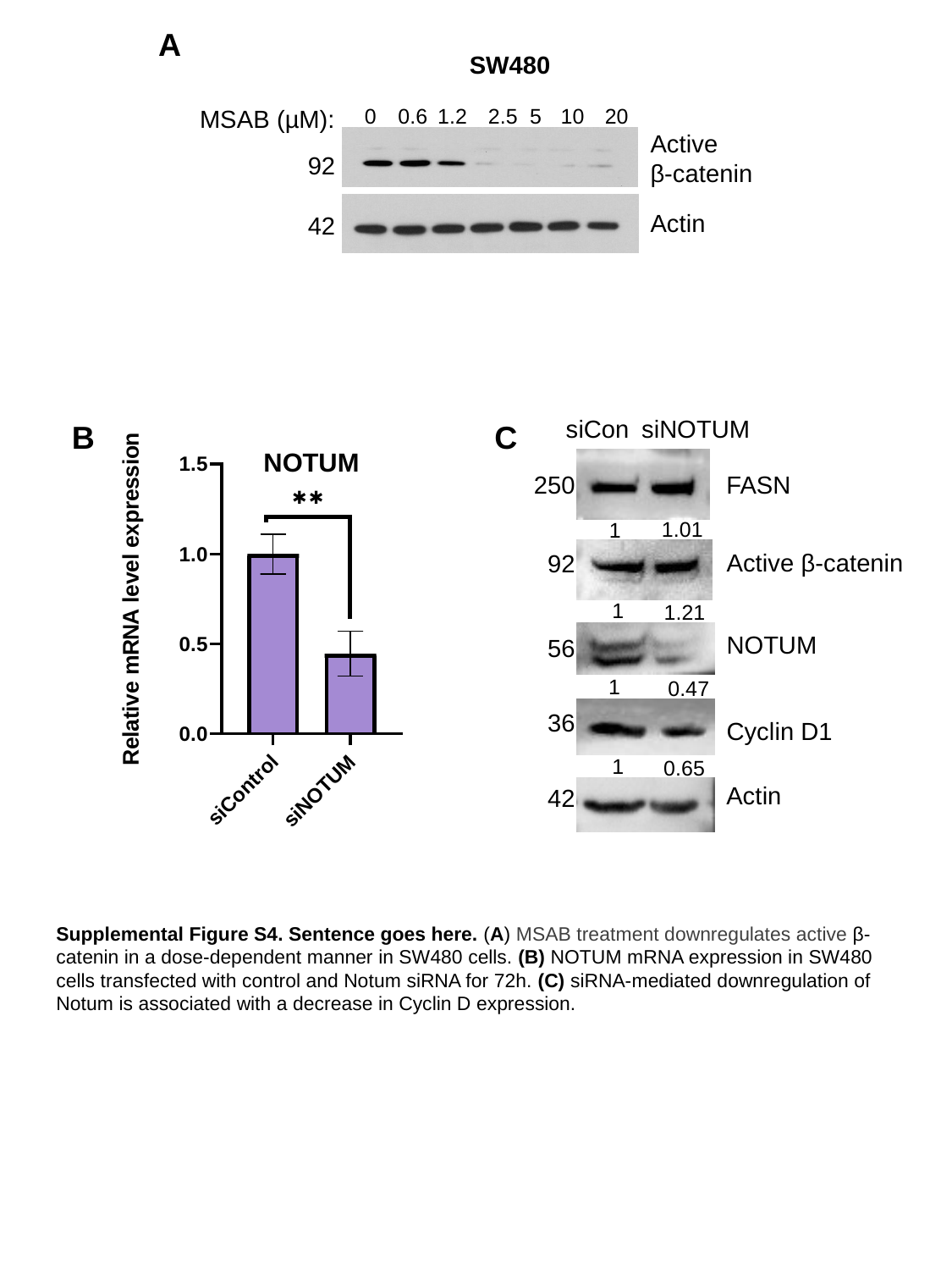

A
SW480
MSAB (µM):
0
0.6
1.2
2.5
5
10
20
Active
β-catenin
92
Actin
42
siCon
siNOTUM
250
FASN
1.01
1
Active β-catenin
92
1
1.21
NOTUM
56
1
0.47
36
Cyclin D1
1
0.65
Actin
42
B
C
Supplemental Figure S4. Sentence goes here. (A) MSAB treatment downregulates active β-catenin in a dose-dependent manner in SW480 cells. (B) NOTUM mRNA expression in SW480 cells transfected with control and Notum siRNA for 72h. (C) siRNA-mediated downregulation of Notum is associated with a decrease in Cyclin D expression.
